# ARRMA: An Integrative Theoretical and Mathematical Model of Assumed and Actual Dyadic Behavior

**DOI:** 10.3389/fpsyg.2022.834796

**Published:** 2022-06-07

**Authors:** Thomas E. Malloy

**Affiliations:** Rhode Island College, Providence, RI, United States

**Keywords:** assumed reciprocity, reciprocity, metaperception accuracy, social relations model, dyadic behavior

## Abstract

In dyadic interaction, do people share a common interpersonal reality? Each assumes the probable response of the other, observes the other’s actual response, and assess the veracity of assumptions. Interpersonal theory stipulates that one’s response invites a similar (e.g., smiling elicits smiling) or a dissimilar (e.g., dominance evokes submission) reciprocal response. Members’ assumptions may be congruent or incongruent with the other’s actual response. A model called ARRMA integrates this dyadic interplay by linking three conceptually and mathematically related phenomena: ***A****ssumed* ***R****eciprocity*, ***R****eciprocity*, and ***M****etaperception* ***A****ccuracy*. Typically studied independently, mathematical derivations reveal the necessity of considering their simultaneity. The theoretical logic of minimal ARRMA models at the individual (i.e., in multiple dyads) and dyadic (i.e., specific dyads) levels are developed, and are then generalized to the full ARRMA at each level. Also specified are statistical methods for estimating ARRMA parameters. ARRMA models shared and idiosyncratic interpersonal realities in dyads.

The human cortex is disproportionally large compared to many animals, and social brain theory provides an explanation; the human cortex evolved to manage the complexity of interpersonal relationships (Dunbar and Shultz, 2007; Dunbar, 2014). ARRMA is an integrative theoretical and mathematical model of the complex dynamic interplay of three interpersonal dyadic phenomena: ***A****ssumed* ***R****eciprocity*, ***R****eciprocity* and ***M****etaperception* ***A****ccuracy*. Assumed reciprocity is A’s belief that B will respond to A as A responds to B, and is an interpersonal assumption. Reciprocity is the congruence, incongruence, or independence of interpersonal responses; is A’s response to B and B’s response to A similar, dissimilar, or independent? Metaperception accuracy is the veridicality of A’s assumptions about how B will respond to A; B’s actual response to A is the validity criterion. Occurring simultaneously in dyadic interaction, ARRMA captures this complex arrangement of assumed and actual interpersonal responses that the human cortex evolved to manage.

Typically, these phenomena have been conceptualized and studied independently (Tagiuri, 1958; Kenny, 1994), although Eisenkraft et al. (2017) studied them simultaneously at the dyadic level (i.e., in specific dyadic arrangements) using a mediational model. They proposed that B achieves metaperception accuracy when A directly communicates thoughts, feelings, or behavioral intentions to B. In the absence of explicit communication, if A’s and B’s responses are reciprocal, either member can achieve accuracy by simply assuming the other reciprocates their thoughts, feelings, or behavioral intentions (i.e., “self-projection” according to Eisenkraft et al., 2017). In a mediational model of interpersonal affect, Eisenkraft and colleagues propose that B’s liking for A causes A’s liking for B, and if A simply projects their liking for B when predicating B’s liking for A, metaperception accuracy is achieved. However, as detailed by Kenny (2020) there is a basic concern with the mediational approach to studying assumed reciprocity, reciprocity, and metaperception accuracy. The mediational models used specify unidirectional causation when, ‘‘In fact, the causation flows in both directions’’ (p. 228). In contrast to the mediational approach, ARRMA specifies that assumed reciprocity, reciprocity, and metaperception accuracy occur simultaneously, and are intertwined theoretically and mathematically at both the individual (i.e., among people in general) and dyadic (i.e., specific dyadic arrangements) levels. The variables in ARRMA are components of the Social Relations Model^1^ (SRM, Malloy, 2018b), and the paths connecting SRM components are estimates of ARRMA phenomena. Figure 1 presents a conceptual model of the SRM. Figures 2, 3 show the ARRMA specifications at the individual and dyadic levels, respectively. In contrast to a mediational model of metaperception accuracy, at both levels there is one endogenous variable caused by two exogenous variables. As shown mathematically, changes in one of the phenomena affects the occurrence of the other two.

**FIGURE 1:**
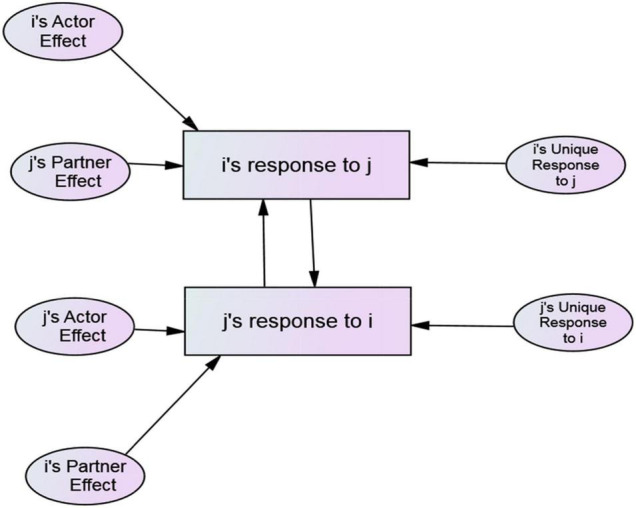
A conceptual representation of the SRM at the individual and dyadic levels.

**FIGURE 2:**
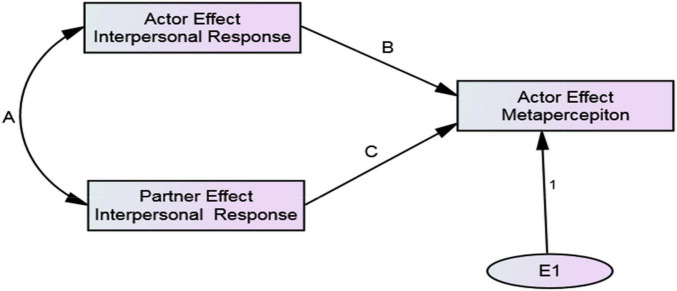
ARRMA model at the individual level of analysis. Parameters B, A, and C are assumed reciprocity, reciprocity, and metaperception accuracy, respectively.

**FIGURE 3:**
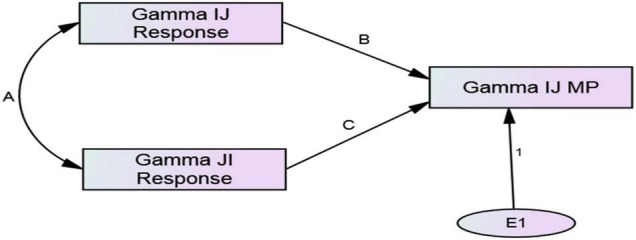
Minimal dyadic ARRMA with incomplete data (gammas are SRM relationship effects).

## A Roadmap

Before considering model details, the conceptual and statistical foundations of ARRMA are established. First, theories of interpersonal behavior provide general guidance on this dynamic interplay of assumptive and actual social behavior, and their impact on the meta-accuracy of social knowing. Second, there is a review of the ARRMA phenomena. Third, I discuss the SRM; its components are variables in ARRMA, and I show how associations of SRM components quantify ARRMA phenomena. Presented are the formal derivations of the minimal ARRMA models at the individual and dyadic levels (Figures 2, 3, respectively), with generalization to the full ARRMA models. Also discussed are statistical implications for ARRMA modeling when members are distinguishable and indistinguishable.

### Theories of Interpersonal Behavior

Theories of interpersonal behavior (Sullivan, 1939, 1949; Leary, 1957; Schutz, 1958; Kiesler, 1996; Kenny, 2020) emphasize the assumptive processes and actual behaviors that occur in dyadic interaction, and their functional significance for coordinated action. Sullivan (1949) argued that people must understand their own and their partner’s behavior, and introduced the concept of “consensual validation” or the “degree of approximate agreement with a significant other person or persons which permit the drawing of generally useful inferences about the action and thought of the other” (p. 177). ARRMA assumes that in a dyadic interaction each member assesses their own behavior and that of the other, so that coordinated adaptive responses can occur. These assessments are central in Leary’s (1957) theory; he proposed that “The interpersonal theory requires that for each variable or variable system by which we measure the subject’s behavior. We must include an equivalent set for measuring the behavior of each specified ‘other’ with whom the subject interacts” (p. 39). A corollary of Leary’s theory is that interpersonal processes are “multilevel” with causal mechanisms at the individual and dyadic levels. Consistent with these principles, the responses of each member of the dyad are measured, and ARRMA models assumed and actual dyadic responses at two levels of analysis—individual and dyadic. A core construct in ARRMA is metaperception (Kenny and DePaulo, 1993); an individual’s prediction of the partner’s response to oneself (Laing et al., 1966), that also occurs at the individual and dyadic levels. Schutz’s (1966) theory of interpersonal behavior invokes similar logic: “An interpersonal situation is one involving two or more persons, in which these individuals take account of each other for some purpose or decision.” (p. 14). To coordinate action, dyad members must share a somewhat common social reality.

Interpersonal theory proposes that in dyadic interactions, the behavior of each member has an impact on the response of the other (Leary, 1957; Carson, 1969), and the failure to consider this bi-lateral impact is a conceptual problem termed *pseudounilaterality* (Duncan et al., 1984). Leary, for example, proposed that “Interpersonal reflexes tend (with a probability significantly greater than chance) to initiate or invite reciprocal interpersonal responses from the ‘other’ person in the interaction that lead to a repetition of the original reflex” (p. 123). These entrained interpersonal responses are one basis for positive (e.g., agreeable responses invite agreeableness) or complementary (e.g., dominance invites submissiveness) reciprocity. Sadler and Woody (2003) provide empirical support for positive and complementary reciprocity for affiliation and dominance, respectively, in mixed-sex dyadic interactions. Each individual is aware of their own perceptions, affect, or intended actions, and their expectation for how the other will respond to them. These expectations, termed metaperceptions, may be veridical or erroneous. Schutz’s (1966) theory of fundamental interpersonal relations orientation (FIRO) offers similar propositions. Positioned in dyadic interaction, FIRO theory proffers that each member has some inclination to express inclusion, control and affection at some level to the other, and that each expects (i.e., wants) a response from the other at some level on each of these dimensions. When dyad members’ expressed and wanted behaviors are commensurate (e.g., A expresses a high level of affection, and B wants a high level) the dyad is interpersonally compatible. When incommensurate, incompatibility is the result. Algorithms that formalize the level of dyadic compatibility specified by FIRO theory are available (Malloy and Copeland, 1980), but do not estimate compatibility at both the individual and dyadic levels.

Thus, interpersonal theory proposes that the behaviors of dyad members are affected simultaneously by the behavioral inclinations of the actor (Malloy et al., 2005), the behavior of the partner (Kenny and Malloy, 1988; Markey et al., 2003) and by the unique responses that specific people make to one another (Kenny and La Voie, 1984). Interpersonal theory (Leary, 1957) also proposes a strain toward reciprocity in dyads because one’s own behavior engenders similar behavior by the other, as well as assumed reciprocity because people seek balanced cognitive representations of social relationships (Heider, 1958). Coupled with reciprocity and assumed reciprocity are expectations about how the other will behave, that can be accurate or inaccurate. These theories converge and bolster the claim that interpersonal assumptions and actual behaviors occur simultaneously in dyadic interactions. ARRMA models this simultaneity and integrates this interplay by focusing on three dyadic phenomena representing assumptive social relationships (assumed reciprocity), actual social relationships (reciprocity), and the accuracy of interpersonal assumptions (metaperception accuracy). Others have recognized that these phenomena co-occur, but have not formally integrated them in a single mathematical model at the individual and dyadic levels (Elfenbein et al., 2009; Eisenkraft et al., 2017). ARRMA is an integrative mathematical model of complex assumed and actual dyadic relationships.

Studying the ARRMA phenomena simultaneously at the individual and dyadic levels offers an important theoretical advance; this approach is justified by Tagiuri’s (1958) claim that “The two-person group is without doubt the most crucial social situation, perhaps even the most crucial of all human situations” (p. 329). In dyads, each member is motivated to assess their own behavior, make assumptions about how the other will behave, and assess the veridicality of interpersonal assumptions (Sullivan, 1949; Leary, 1957; Schutz, 1966). Cognitive theory provides mechanisms that operate when making these assessments. Specifically, an ***availability-balance model*** (Malloy, 2018a,^2019^) is used to understand processes that occur in dyads when each member makes interpersonal judgments, assumptions, and metaperceptions.

### An Availability-Balance Model of Interpersonal Knowing

Upon encountering a stranger, base rate assumptions about people in general lead to interpersonal expectations. Attention must be allocated to assess the other, anticipate how the other will behave, and coordinate interpersonal responses. This is very taxing on the social cognitive systems of information processing misers (Fiske and Taylor, 1991). So, how do people simplify this complexity? In a dyadic interaction, one readily available source of information is one’s own thoughts, feelings and behavioral intentions toward others generally, and toward specific others. Person A may strive for, and expect, congenial interactions with most others, while acknowledging specific others with whom A behaves uniquely and unpleasantly. Repeated interactions with a person provides a large sample of interpersonal behavior, and the perceiver assumes that past responses are likely to occur again, and that established reciprocal behavior of the past will probably be reenacted in the present. These are assumptions about a specific person, but when aggregated across interaction partners, they are nomothetic assumptions about people generally. Yet, these assumed and actual social responses occur simultaneously at the individual and dyadic levels. The individual level is concerned with the consistency of a person’s responses to multiple others, and their responses to the person. Yet as demonstrated by almost 40 years of social relations modeling (Malloy, 2018b; Kenny, 2020), interpersonal relationships also operate at the dyadic level. The dyadic level is concerned with unique responses that people make to specific others. Because social behavior unfolds simultaneously at the individual and dyadic levels, failure to conceptualize and estimate phenomena at both levels is a conceptual error that confounds them (Kenny and Nasby, 1980).

### ARRMA Research

Only recently has the ARRMA model been used empirically. Among very highly acquainted (some for decades) family members, friends, and co-workers, ARRMA provided evidence of assumed reciprocity, reciprocity and metaperception accuracy of attraction at the individual level (Malloy, 2018a). People assumed that others were as attracted to them as they were attracted to those others and, in fact, there was actual reciprocity of attraction although it was much weaker than assumed reciprocity. There was also evidence for metaperception accuracy at the individual level; people knew how attracted to them members of these groups actually were. Although dyadic assumed reciprocity was robust, dyadic reciprocity and metaperception accuracy were weaker.

Family members, friends and co-workers also judged the similarity of the members of these groups to themselves. ARRMA produced reliable estimates of assumed reciprocity, reciprocity, and metaperception accuracy for similarity judgments at the individual level in all of them (Malloy, 2019). Moreover, in these groups there was dyadic assumed reciprocity, whereas dyadic reciprocity and metaperception accuracy were observed only among family members when judging specific others’ similarity to themselves. Empirical evaluation of ARRMA suggests that assumed reciprocity, reciprocity, and metaperception accuracy may be most strongly operative in relationships with people generally. Counterintuitively, in long term relationships with specific others that are unlikely to change, at least in the short run, assessments of specific others seem to be dominated by one’s assumptive world. I enthusiastically crave spicy Szechuan Chinese food for dinner and assume my friend will be similarly inclined (assumed reciprocity), but when she is unenthusiastic (non-reciprocity), I recognize my assumptive error regarding her attitude (metaperception inaccuracy). An alternative dinner plan that satisfies both is the likely outcome. In this case, actual social responses confront interpersonal assumptions incompatible with them. ARRMA captures the dyadic interplay of interpersonal assumptions, actual responses, and the veridicality of one’s assessment of how the other will behave.

The implementation of the Social Relations Model (SRM) for dyadic data (Malloy, 2018b), and the integration of relevant SRM components in the specification of ARRMA phenomena, demonstrates they are conceptually and statistically bound at the individual and dyadic levels. Empirically assessing one of the phenomena while ignoring the other two is imprecise theoretically, and yields biased estimates of the single phenomena because they are a theoretical and empirical triad. The present approach to ARRMA phenomena requires an explicit recognition of the componential structure of interpersonal responses (Malloy and Albright, 1990), and the SRM (Kenny and La Voie, 1984; Malloy and Kenny, 1986; Bond and Malloy, 2018) guides this necessary first step. After the initial social relations analysis, the relevant components of dyadic scores at the individual and dyadic levels become variables in the ARRMA model. Finally, estimates of the model’s parameters linking SRM components quantifies the three phenomena comprising the ARRMA triumvirate.

## The ARRMA Triumvirate

### Assumed Reciprocity

Assumed reciprocity has received attention for at least 60 years (Tagiuri, 1958; Rodgers, 1959), and was discovered serendipitously in the mid-20th century by investigators interested in the accuracy of interpersonal perception (Fiedler, 1954; Cronbach, 1955). In a typical study, an individual responded to personality test items and then predicted how another person would respond to those items. One’s own responses to the items and the predicted responses to them by another are correlated with item as the unit of analysis. Cronbach (1955) critiqued these accuracy correlations and deemed them inadequate because interpersonal judgments have a meaningful componential structure that must be partitioned, and only relevant components correlated to estimate accuracy (cf. Kenny and Albright, 1987). This same logic holds for ARRMA. Although individual differences in accuracy were elusive empirically, a serendipitous discovery was that people showed a robust inclination to “assume similarity” with others. That is, people assumed that others would respond to test items as they did. This initial discovery has been replicated in different laboratories investigating different phenomena in social and personality psychology (e.g., Cronbach, 1955; Rodgers, 1959; Eiser and Taylor, 1972; Kenny, 1994; Watson et al., 2000; Lee et al., 2009; Human and Biesanz, 2011; Paunonen and Hong, 2013). People assume that others think, feel, and act as they do.

Theoretical analysis (Laing et al., 1966) shifted the focus from A’s unidirectional assumption that B would respond as A did, to the meta-personal in which A predicts how B will respond to A. This recalibration shifted attention to assumed reciprocity that occurs when people predict that others think, feel, and act toward them as they think, feel, and act toward those others. Tagiuri (1958, p. 321) was interested in assumed reciprocity of social preferences (i.e., attraction) and reported “the tendency … to perceive a person’s feeling for us as congruent with our feelings for him,” and observed that it “exceeds what would be expected on the basis of actual levels of reciprocation.” Tagiuri clearly had insight into some aspects of ARRMA. Kenny (1994) echoed Tagiuri’s conclusion, and a meta-analytic synthesis of research on trait perception and affect in dyads led him to the conclusion that “Assumed reciprocity correlations are some of the largest correlations in interpersonal perception” (p. 103).

The availability-balance model (Malloy, 2018a,b) provides a parsimonious theoretical account of assumed reciprocity. When assessing how another will respond to oneself (Leary, 1957), self-assessed behavior is available cognitively, and serves a heuristic anchoring function when predicting the other’s response (Tversky and Kahneman, 1973, 1974; Park et al., 1994). These predictions can conform to rational statistical models in which population base rates are the basis for predictions. Mere knowledge that most people are not violent can be the basis for predicting accurately how a randomly selected person from the population is likely to respond to me. However, as Kahneman and Tversky (1973) demonstrated, when people have available, seemingly diagnostic information deemed relevant to an inference task, base rates are ignored in favor of that information. Cognitive representations of one own characteristics (i.e., diagnostic cues) serve this purpose, and lead people to infer that the other’s response to them will match their response to the other. Whether the other is a stranger, an acquaintance, or someone with whom one has a long-term relationship, there is motivation to assume reciprocal, balanced interpersonal processes (Heider, 1958). Balanced interpersonal assumptions are less taxing cognitively because one need only consider one’s attitudes and intentions toward the other, and then assume reciprocity. In dyadic encounters, heuristically available cognitions, affect, and behavioral intentions (Tversky and Kahneman, 1973, 1974; Park et al., 1994) are assumed to be reciprocated by people in general, and by specific others (Leary, 1957) thereby producing balanced interpersonal systems (Heider, 1958). That is, assumed reciprocity is a mechanism people use to achieve interpersonal balance. Assumed reciprocity occurs in one’s responses to multiple others (i.e., the generalized individual level), and in one’s responses to specific others (the dyadic level). Assumed reciprocity, one of the strongest phenomena in interpersonal perception, is a consequence of the motivation to perceive balance and symmetry in cognitive representations of social relationships. Assuming balance, even when wrong, is less disquieting than assuming imbalance. This availability-balance principle can explain why assumed reciprocity is such a robust, ubiquitous phenomenon.

### Reciprocity

Reciprocity is a universal law of social behavior (Gouldner, 1960) and a basis for moral codes (e.g., do unto others as you would have them do unto you), rational systems of law, and economics (Fukuyama, 1996). Reciprocity also serves an adaptive function among humans. In infancy, humans are incapable of surviving independently and must rely on mature adults to nurture them. The adult human face affords visual attention for young, vulnerable children (McArthur and Baron, 1983), and infants show a spontaneous preference for human faces at birth (Morton and Johnson, 1991). Infants direct visual to human faces more than to those of other primates (Sanefuji et al., 2014). Parents know the joy and impulse to nurture when their infant gazes into their eyes. When most vulnerable, infants’ preferences for human faces compared to non-faces, or faces of other primates, is an inherently dyadic response serving an adaptive function because it promotes reciprocal nurturance by mature adults. In caregiver-infant dyads, evolved interpersonal mechanisms operate that promote reciprocal responses, bonding, and survival of the immature. For example, the distressed infant’s cry is inherently averse for an adult, and motivates behavior to remove the source of distress, sooth the child, and stop the crying. These reciprocal interpersonal responses promote secure dyadic attachment (Shaver and Mikulincer, 2012). In an infant-adult dyad, the distressed infant’s crying is reinforced positively, and the adult responsiveness and soothing is reinforced negatively. This reciprocal dyadic entrainment increases the likelihood of infant survival, and even has implications for the infant’s relationship formation and maintenance later in life (Shaver and Mikulincer, 2014).

Reciprocity also serves other adaptive functions throughout the lifespan. With development people learn cultural criteria that define social status in the same-sex peer group; that is, one’s relative rank in the status hierarchy. Reciprocal attraction and relationship formation is most likely with members of the opposite sex whose status ranking is similar to one’s own. This assortative mating is evident among humans on attractiveness (Hunt et al., 2015) and religious preference (McClendon, 2016), and among non-humans for physical size (Ng and Williams, 2014). Reciprocal attraction based on attitude and behavioral similarity is an important determinant of relationship formation (Berscheid and Hatfield-Walster, 1969), and operates similarly in long term relationships (Malloy, 2018a). Moreover, married couples have similar risk taking attitudes and the longer the marriage the more similar these attitudes become (Bacon et al., 2014). Males and females who believe there is reciprocity for positive behavior (“If I do nice things for someone, I can anticipate that they will respect me and treat me just as well as I treat them.”) live longer lives than those who do not make this endorsement (Skrabski et al., 2004). Interpersonal responses to in-group members are typically more favorable than those to out-group members because people assume that in-group members will reciprocate favorability (Gaertner and Insko, 2000). Reciprocity is an adaptive law of social behavior, and is a foundation principle of systems that assume human rationality and fairness (e.g., economics, law, and ethical codes).

### Metaperception Accuracy

Metaperception accuracy is the third phenomenon of the ARRMA triumvirate. In dyads, people are aware of their thoughts, feelings, and behavioral intentions toward the other while, at the same time, spontaneously consider others’ thoughts, feelings, and behavioral intentions toward them. These assessments of others responses to the self by people in general, and by specific people, are metaperceptions at the individual and dyadic levels, respectively. When adults are asked to predict others’ trait judgments of them (Kenny, 1994) or interpersonal attraction to them (Malloy, 2018a) these predictions are accurate beyond chance. When adolescents (Malloy and Cillessen, 2008) and children (Malloy et al., 2007) predicted peers’ judgments of their academic ability, metaperception accuracy was observed; even among those 6 years old. Well acquainted college students living in dormitory suites were accurately aware of how suitemates generally, and specific suitemates, judged their personality traits (Malloy and Albright, 1990). Even when behavior toward the other was scripted experimentally, thus being independent of one’s dispositional inclinations, undergraduates were able to accurately predict how the other judged them as they followed the behavioral script provided by the researchers (Albright et al., 2001). Kenny (1994) reported that the median metaperception accuracy correlation for trait judgments at the individual level of analysis was substantial (*r* = 0.58), and in five studies of interpersonal liking, Kenny reported an average metaperception accuracy correlation of *r* = 0.47. Among highly acquainted family members, friends and co-workers, ARRMA revealed statistically reliable generalized metaperception accuracy among family members on task attraction and physical attractiveness, among friends on physical attractiveness, and among co-workers on task attraction (Malloy, 2018a). Members of these groups were very highly acquainted, and dyad members were accurately aware of others attraction to them on some dimensions.

### Interim Summary

Behavioral scientists have studied assumed reciprocity (Tagiuri, 1958; Rodgers, 1959), reciprocity (Gouldner, 1960; Newcomb, 1979) and metaperception accuracy (Kenny, 1994) as separate phenomena for decades. Realization that they are bound inextricably theoretically and statistically, led to the specification of ARRMA. Research that has implemented the ARRMA model was focused on interpersonal attraction (Malloy, 2018a) and self-referenced perceived interpersonal similarity (Malloy, 2019). Because the ARRMA phenomena are relevant in any dyad, the model can refine understanding of the assumptive and actual responses operative in different social contexts (e.g., variation in acquaintance or the nature of a relationship), and for different classes of social behavior (e.g., trait judgments, interpersonal affect, overt behavior).

Because dyadic behavior has a theoretically meaningful componential structure (Warner et al., 1979; Kenny, 1994, 2020; Bond and Malloy, 2018; Malloy, 2018b), a necessary first step in the estimation of ARRMA parameters is an initial social relations analysis. That initial analysis partitions dyadic scores into actor (or perceiver), partner (or target), and relationship (or uniqueness) components. The appropriate components of dyadic responses then become variables in the specification of the ARRMA model at the individual and dyadic levels. Parameter estimation follows and estimates the strength of co-occurring ARRMA phenomena. I first consider social relations analysis; then the logic and formal derivations of ARRMA are developed. Following this, the estimation of ARRMA parameters is extended to distinguishable (e.g., a mixed-sex) and indistinguishable (e.g., same-sex) dyads.

## Social Relations Analysis: A Precursor to ARRMA

Estimation of ARRMA parameters at the individual and dyadic levels requires an initial social relations analysis. Consider dyad members *i* and *j* who rate their interpersonal attraction to one another on a 9 point scale (1 = lowest attraction and 9 = highest attraction). Assume that *i*’s rated attraction to *j* is an 8 and *j*’s rated attraction to *i* is a 4. These are whole, undecomposed attraction ratings that naively index *i*’s and *j*’s interpersonal attraction. However, since Cronbach’s (1955) seminal insight into the componential structure of interpersonal responses, the complex structure lurking within these scores must be acknowledged and modeled. Each dyadic score contains distinct components of interpersonal responses that have different psychological meaning. The social relations model (Warner et al., 1979; Kenny and La Voie, 1984; Malloy and Kenny, 1986; Bond and Malloy, 2018; Malloy, 2018b; Kenny, 2020) is a formal specification of the components of dyadic responses at the individual and dyadic levels. Group effects are also estimable (e.g., Kenny et al., 2006; Cook, 2015) and, in principle, could be modeled with ARRMA although this is beyond the present scope. The key point is that the simultaneous estimation of the ARRMA parameters at the individual and dyadic levels first requires a partitioning of un-decomposed dyadic scores into theoretically meaningful components. Social relations analysis accomplishes this partitioning. Then, the relevant components of the SRM become terms in the ARRMA model. Consider the components specified by the SRM.

### Social Relations Model Components

The SRM specifies that interpersonal responses in dyads contain three theoretically meaningful components. Those components are the actor effect (α), the partner effect (β), and the relationship effect (γ). To estimate these effects, a ***multiple interaction dyadic design*** (people interaction with multiple partners in dyads) that yields sufficient data for the decomposition of whole scores into SRM components must be used (Malloy, 2018b). The designs most likely used prior to ARRMA modeling are the asymmetric block and the round robin. In the asymmetric block design, members of one category (e.g., an in-group) interact with members of another category (e.g., an out-group); within-category responses do not occur. Members of different categories also predict how each member of the other category will respond to them. In a round robin, randomly constituted members of a group (minimally 4) respond to all other members of the group. Each member of every dyad makes reciprocal interpersonal responses, and for purposes of ARRMA would predict (i.e., metaperceptions) how their partner responded to them. These metaperceptions could reference a partner’s trait rating, affective response, or interpersonal behavior. Actor, partner, and relationship components of the SRM are not estimable from a ***single interaction dyadic design*** where *i* and *j* respond only to one another (Malloy and Albright, 2001). If a single interaction design without social relations analysis is used to estimate ARRMA parameters, those estimates would confound individual and dyadic effects (cf. Kenny and Nasby, 1980). To maximize the yield of ARRMA modeling, a multiple interaction design and componential social relations analysis is the best course. The actor, partner, and relationship components computed in the social relations analysis then the variables in ARRMA models.

The SRM ***actor effect*** quantifies consistent individual differences in actors’ behaviors when interacting with the same partners. For example, one person may consistently smile at others, whereas another may rarely smile at them. The ***partner effect*** quantifies individual differences in behavior elicited by different partners when interacting with the same actors. For example, one partner may consistently elicit smiles from the actors with whom she interacts, whereas another consistently elicits less smiling. Actor and partner effects index the consistency of behavioral responses of one to many (i.e., actor) and many to one (i.e., partner). Statistically, individual is the unit of analysis. The ***relationship effect*** is at the level of the dyad and quantifies one person’s unique behavioral response to a specific partner while controlling their respective actor and partner effects, and dyad is the unit of statistical analysis. Figure 1 provides a conceptual model of actor, partner, and relationship effects at the individual and dyadic levels.

The conceptual model in Figure 1 depicting *i*’s response to *j* (*X*_*ij*_) can be represented by the SRM theoretical equation whose terms are latent variables estimated with data:


(1) $$X_{ijk}=\mu_{k}+\alpha_{ik}+\beta_{jk}+\gamma_{ijk}+\varepsilon_{ijk}$$

Equation 1 states that in group *k* (e.g., round robin, an asymmetric block), *i*’s response to *j* (*X*_*ijk*_) is equal to the average level of behavior *X* in group *k* (μ*_*k*_*), plus the consistency of *i*’s responses to the members of group *k*, including *j* (i.e., *i*’s actor effect α*_*ik*_*), plus the effect that *j* has on the behavior of the members of *k*, including *i* (i.e., *j*’s partner effect β*_*jk*_*), plus *i*’s unique response to *j* in group *k* (i.e., relationships effect γ*_*ijk*_*) while controlling α*_*ik*_* and β*_*jk*_*. Random error is also present in *i*’s response to *j* in group *k* (i.e., ε*_*ijk*_*).

Represented in equation 2 is the reciprocal response of *j* to *i* on variable *X* with a change in subscripts that represent *j* as an actor, *i* as a partner, and *j*’s unique response to *i*. That is:


(2) $$X_{jik}=\mu_{k}+\alpha_{jk}+\beta_{ik}+\gamma_{jik}+\varepsilon_{jik}$$

Equations 1 and 2 are the theoretical SRM equations that specify the presumed components of dyadic scores. Equations 1 and 2 meet Leary’s (1957) call for simultaneous measurement of interactants responses at multiple levels of analysis (i.e., group, individual, and dyad).

### Social Relations Model Components in the Round Robin Design

Table 1 illustrates the structure of a round robin design depicting interpersonal responses (*r*’s) and metaperceptions (*mp*’s) with subscripts defining the row (actor) and column (partner) for dyad members. Computation of SRM actor components in a round robin follows the specification of Warner et al. (1979) that accommodates the missing diagonal elements (i.e., self-data). The computation of the actor component (α) for person A across multiple interactions (α***_1._****)* is:


(3) $${\text{α}}_{1.}=\begin{array}{c} {\left(n-1\right)}^{2}\\ ----\\ \left(n^{2}-2n\right) \end{array}M_{1.}+\begin{array}{c} \left(n-1\right)\\ ----\\ \left(n^{2}-2n\right) \end{array}M_{.1}\;-\;\begin{array}{c} \left(n-1\right)\\ ----\\ \left(n-2\right) \end{array}M..$$

**TABLE 1:** Multiple interaction designs for ARRMA estimation.

Reciprocal round robin									

Interpersonal responses					Metaperceptions				
**partners**					**partners**				
Actors	A	B	C	D	Actors	A	B	C	D
*A*	–	*r*_12_	*r*_13_	*r*_14_	*A*	–	*mp*_12_	*mp*_13_	*mp*_14_
*B*	*r*_21_	–	*r*_23_	*r*_24_	*B*	*mp*_21_	–	*mp*_23_	*mp*_24_
*C*	*r*_31_	*r*_32_	–	*r*_34_	*C*	*mp*_31_	*mp*_32_	–	*mp*_34_
*D*	*r*_41_	*r*_42_	*r*_43_	–	*D*	*mp*_41_	*mp*_42_	*mp*_43_	–

*r’s are reciprocal interpersonal responses, mp’s are reciprocal metaperceptions.*

*– diagonal elements are not collected.*

In equation 3, *n* is the number of rows (i.e., actors) and is equal to the number of columns (i.e., partners). Terms *M*_1._ and *M*_.1_ are row and column marginal means, respectively. *M*. is the grand mean for the *n*^2^ – *n* (the missing diagonal elements are subtracted) dyadic scores in group *k*. Consider the logic of equation 3. An actor’s average response to multiple partners and the average response of those partners to the actor are weighted by the number of rows and columns, and are then pooled. Subtracted from this sum is the weighted grand mean. The sum of actor and partner components will equal zero because least squares estimation theory is the basis for their computation. The computation of A’s partner component (β) indexing the responses elicited from multiple partners (β_.1_) uses equation 4, and the logic mimics that seen in Equation 3.


(4) $${\text{β}}_{.1}=\begin{array}{c} {\left(n-1\right)}^{2}\\ ----\\ \left(n^{2}-2n\right) \end{array}M_{.1}+\begin{array}{c} \left(n-1\right)\\ ----\\ \left(n^{2}-2n\right) \end{array}M_{1.}\;-\;\begin{array}{c} \left(n-1\right)\\ ----\\ \left(n-2\right) \end{array}M..$$

The computation of an actor’s unique response to a particular partner in equation 5 entails subtracting the actor component, the partner component, and the grand mean from the appropriate element within the round robin matrix. This isolates the relationship component (γ). For example, 1’s unique response to 2 (γ_12_) is computed by:


(5) $$\gamma_{12}=X_{12}-\alpha_{1.}-\beta_{.2}-M..$$

Equation 5 shows that for element *X*_12_ within the round robin, one subtracts the appropriate row (actor) and column (partner) components, and the grand mean. When applied to all elements of the round robin, equation 5 yields the *n*^2^ *-n* reciprocal relationship components. Elements above the diagonal are actors’ unique responses to multiple partners, and those below are partners’ unique responses to multiple actors. Relationship components are yoked within dyads.

### Social Relations Model Components in Blocks of the Asymmetric Block Design

When members of two different categories interact, the asymmetric block design is used. When actors and partners from two different categories (e.g., in-group and out-group) interact, their reciprocal responses are recorded. If members of one category (e.g., in-group) predict out-group partners’ responses to them (i.e., metaperceptions), but members of the other group (e.g., out-group) do not make these predictions, metaperceptions are unidirectional rather than reciprocal. Supplementary Table 1 presents this data structure. Described below is the computation of actor components in in-group members’ responses to out-group members. Also described is the computation of in-group members’ predictions of out-group members’ responses to them. This illustrates the partitioning of SRM components within blocks of the asymmetric block structure.

Consider in-group members’ responses and metaperceptions. Within each block, the grand mean (M.) is the average of the 16 responses and metaperceptions when the 4 in-group actors respond to, and predict, the interpersonal response to them by each of the 4 out-group partners. Then, row means for the four in-group actors in Supplementary Table 1 are computed yielding M_1._, M_2._, M_3._, and M_4._; the subscript indicates the actor (i.e., 1, 2, 3, and 4), and the point (i.e.,.) indicates this is an average across rows (i.e., partners). The column means for each of the four out-group partners computed across columns (i.e., actors) in Supplementary Table 1 yield M_.1_, M_.2_, M_.3_, and M_.4_. Using marginal row (actors) and column (partners) means as well as the grand mean, SRM components can be computed.

An estimate of person *i*’s actor component is:


(6) $$\alpha_{i}=M_{1.}-M..$$

The partner component for each of the out-group members on the columns is computed, and an estimate of *j*’s partner component is:


(7) $$\beta_{j}=M_{.j}-M..$$

Actor and partner components of interpersonal responses and actor components of metaperceptions are the necessary data for estimation of ARRMA parameters at the individual level of analysis. In this example, complete data are available only for the in-group because measurements of the out-group metaperceptions were not taken.

For dyadic ARRMA, *i*’s and *j*’s unique interpersonal responses within dyads (i.e., γ*_*ij*_* and γ*_*ji*_*), as well as *i*’s prediction of *j*’s unique response to *i* are the necessary data for estimation of the parameters of the minimal dyadic ARRMA. The full dyadic ARRMA introduces additional complexity elaborated later. An estimate of *i*’s unique response to *j* applied to interpersonal responses and metaperceptions is produced by:


(8) $$\gamma_{ij}=Y_{ij}-\left(M_{1.}-M_{.}\right)-\left(M_{.j}-M_{.}\right)-M..$$

that reduces to:


(9) $$\gamma_{ij}=Y_{ij}-\alpha_{i}-\beta_{j}-M..$$

SRM components computed in a round robin are random effect estimates (Searle et al., 1992) because groups of actors and partners are constituted randomly. Consequently, these designs meet the standards of representative design, and statistical estimates generalize to the population of actors and partners (Brunswik, 1956). Because this is true of the SRM, it is also true of ARRMA.

## Minimal ARRMA Derivations at the Individual and Dyadic Levels

### ARRMA Parameters at the Individual Level

ARRMA derivations follow standard covariance algebra informed by the tracing rule for structural models (Kenny, 1979), and estimates conform to criteria of least squares estimation theory. The ARRMA model at the individual level of analysis presented in Figure 2 is specified formally by:


(10) $$\alpha_{mp}=b\alpha_{r}+c\beta_{r}+e$$

Equation 10 is just-identified; all parameters of the ARRMA model can be estimated if actor and partner effect in interpersonal responses are measured, along with the actor effect in metaperceptions. However, model fit cannot be assessed because there are no residual degrees of freedom. In Equation 10, α*_*mp*_* is the actor component of a metaperception, α*_*r*_* is the actor component of an interpersonal response, and β*_*r*_* is the partner component of an interpersonal response. Coefficients *b* and *c* estimate assumed reciprocity and metaperception accuracy, respectively, and e is random error. Equation 11 estimates assumed reciprocity.


(11) $$\rho \alpha_{r}\alpha_{mp}=b+c\left(\rho \alpha_{r}\beta_{r}\right)+e$$

Equation 11 states that ρα*_*r*_*α*_*mp*_* is the population correlation of actor effects in an interpersonal response (α*_*r*_*) and actor effects in metaperceptions (α*_*mp*_*), and that ρα*_*r*_*β*_*r*_* is reciprocity. Variables in Equation 11 were defined previously.

Metaperception accuracy at the individual level is defined theoretically by Equation 12.


(12) $$\rho \beta_{r}\alpha_{mp}=c+b\left(\rho \alpha_{r}\beta_{r}\right)+e$$

In Equation 12, ρβ*_*r*_*α*_*mp*_* is the population correlation of partner components of interpersonal responses (β*_*r*_*) and actor components of metaperceptions (α*_*mp*_*). Reciprocity is the population correlation of α*_*r*_* and β*_*r*_*, that is, ρα*_*r*_*β*_*r*_*. The product of ρα_*r*_β_*r*_ and equation 12 yields Equation 13.


(13) $$\left(\rho \alpha_{r}\beta_{r}\right)\left(\rho \beta_{r}\alpha_{mp}\right)=c\left(\rho \alpha_{r}\beta_{r}\right)+b\left(\rho \alpha_{r}\beta_{r}^{2}\right)+e$$

The difference between equations 11 and 13 by subtraction yields Equation 14.


(14) ραrαmp-(ραrβrρ*βrαmp)=b-b(ραrβr2)+e

When Equation 14 is re-expressed, a solution for parameter *b* (assumed reciprocity) is provided by Equation 15.


(15) $$b=\frac{\rho \alpha_{r}\alpha_{mp}-\left(\rho \alpha_{r}\beta_{r}\right)\left(\rho \beta_{r}\alpha_{mp}\right)}{1-{\left(\rho \alpha_{r}\beta_{r}\right)}^{2}}$$

Equation 15 shows that the impact of one’s actor component in a dyadic response on one’s actor component of a metaperception is equal to assumed reciprocity minus the product of reciprocity and metaperception accuracy, divided by 1 minus squared reciprocity.

An estimate of metaperception accuracy at the individual level of analysis (parameter c) is provided by Equation 16.


(16) $$c=\frac{\rho \beta_{r}\alpha_{mp}-\left(\rho \alpha_{r}\beta_{r}\right)\left(\rho \alpha_{r}\alpha_{mp}\right)}{1-{\left(\rho \alpha_{r}\beta_{r}\right)}^{2}}$$

Equation 16 shows that the impact of the SRM partner component on the actor component of a metaperception is equal to the accuracy of metaperception, minus the product of reciprocity and assumed reciprocity. This product is then divided by 1 minus squared reciprocity.

These specifications demonstrate that the ARRMA triumvirate are conceptually and statistically intertwined at the individual level of analysis. Estimating any one while ignoring the others is likely to yield biased, unreliable estimates.

### ARRMA Parameters at the Dyadic Level

The derivation of the minimal dyadic ARRMA model (Figure 3) follows logic that is similar to the model at the individual level, but the variables are relationship components (called gamma) with actor and partner components extracted. The dyadic ARRMA specified in Equation 17 is the minimal specification of the model, and requires *i*’s unique response to *j* (γ*_*ij*_*) and *j*’s unique response to *i* (γ*_*ji*_*) from a preliminary social relations analysis. In addition, *i*’s prediction of *j*’s unique response to *i* (a dyadic metaperception) is also required. Discussed later is a generalization of this minimal specification to the full dyadic ARRMA.


(17) $$\gamma_{imp,ji}=\left(b\right)\gamma_{ij}+\left(c\right)\gamma_{ji}+e$$

Equation 17 specifies that γ*_*imp,ji*_* is the relationship component of *i*’s metaperception (i.e., prediction) of *j*’s response to *i*. The term γ*_*ij*_* is *i*’s unique response to *j* on a dyadic variable, and γ*_*ji*_* is *j*’s unique response to *i* on that same variable. Coefficients *b* and *c* quantify assume reciprocity and metaperception accuracy at the dyadic level. Random error is represented by e. Assumed reciprocity at the dyadic level is specified by Equation 18.


(18) $$\rho \gamma_{imp,ji}\gamma_{ij}=b+c\left(\rho \gamma_{ij,}\gamma_{ji}\right)+e$$

Equation 18 states that the population correlation (ρ) of *j*’s unique response to *i* and *i*’s metaperception of *j*’s unique response to *i* is a function of dyadic assumed reciprocity (parameter *b*) plus the product of metaperception accuracy (parameter *c*) and dyadic reciprocity [ργ*_*ij*,_*γ*_*ji*_*]. Dyadic metaperception accuracy (parameter *c*) is specified by Equation 19.


(19) $$\rho \gamma_{ji,}\gamma_{imp,ji}=c+b\left(\rho \gamma_{ij,}\gamma_{ji}\right)+e$$

The correlation of γ*_*ij*_* and γ*_*ji*_*, that is, ργ*_*ij*,_*γ*_*ji*_*, is dyadic reciprocity. The product of ργ*_*ij*,_*γ*_*ji*_* (dyadic reciprocity) and Equation 19 yields Equation 20; that is:


(20) $$\left(\rho \gamma_{ji,}\gamma_{imp,ji}\right)\left(\rho \gamma_{ij,}\gamma_{ji}\right)=c\left(\rho \gamma_{ij,}\gamma_{ji}\right)+b\left(\rho \gamma_{ij,}\gamma_{ji}^{2}\right)+e$$

By subtraction, the difference between Equations 19 and 20 yields Equation 21:


(21) (ργij,γimp,ji)-(ργij,γjiρ*γji,γimp,ji)=b-b(ργij,γji2)+e

When re-expressed, Equation 21yields a solution for parameter *b* (assumed dyadic reciprocity), that is provided by Equation 22.


(22) $$b=\frac{\left(\rho \gamma_{ij,}\gamma_{imp,ji}\right)-\left(\rho \gamma_{ij,}\gamma_{ji}\right)\left(\rho \gamma_{ji,}\gamma_{imp,ji}\right)}{1-{\left(\rho \gamma_{ij,}\gamma_{ji}\right)}^{2}}$$

Equation 22 states that the impact of *i’*s relationship component in response to *j* on *i*’s relationship component of a dyadic metaperception with *j* (i.e., prediction of *j*’s response to *i*) is equal to assumed dyadic reciprocity, minus the product of dyadic reciprocity and dyadic metaperception accuracy, divided by 1 minus squared dyadic reciprocity. If dyadic assumed reciprocity is stronger than dyadic reciprocity, parameter *b* should be a substantial determinant of dyadic metaperception.

Dyadic metaperception accuracy (i.e., parameter *c*) is estimated by Equation 23.


(23) $$c=\frac{\rho \gamma_{ij,}\gamma_{imp,ji}-\left(\rho \gamma_{ij,}\gamma_{ji}\right)\left(\rho \gamma_{ji,}\gamma_{imp,ji}\right)}{1-{\left(\rho \gamma_{ij,}\gamma_{ji}\right)}^{2}}$$

Equation 23 specifies that the effect of *j*’s dyadic relationship component in response to *i* on *i*’s dyadic metaperception of *j*’s response to *i*, is equal to dyadic metaperception accuracy (ργ*_*ji*,_*γ*_*imp*,ji_*) minus the product of dyadic reciprocity and dyadic assumed reciprocity [(ργ*_*ij*,_*γ*_*ji*_*) (ργ*_*ij*,_*γ*_*imp,ji*_)*], that is then divided by 1 minus squared dyadic reciprocity [1 – (ργ*_*ij*,_*γ*_*ji*_*)^2^].

This dyadic specification again shows that the ARRMA phenomena are conceptually and statistically intertwined; estimates of one while ignoring the others will yield biased and unreliable estimates of these dyadic phenomena.

## Empirical Estimation of ARRMA Parameters

A social relations analysis produces estimates of the theoretical parameters of equations 1 and 2. Minimal ARRMA analysis at the individual and dyadic levels is straightforward when using path or structural equation modeling. The full dyadic ARRMA presents greater analytic challenges. Consider each in turn.

### ARRMA Parameter Estimation at the Individual Level of Analysis

ARRMA parameters are estimable at the individual level of analysis when there is sufficient information to estimate assumed reciprocity, reciprocity, and metaperception accuracy following an initial social relations analysis. A round robin design is probably the most common structure used for estimation of ARRMA at the individual level, and example data are presented in Table 1. The reciprocal interpersonal responses (i.e., *r*’s) and metaperceptions (i.e., *mp*’s) each form a round robin that can be analyzed with applications that compute and output actor and partner components (called effect estimates) such as Soremo (Kenny and Xuan, 2004) or TripleR (Schönbrodt et al., 2012). The social relations analysis will produce actor and partner components of interpersonal responses and metaperceptions for each individual. Though produced, partner components in metaperceptions are irrelevant for ARRMA at this level. However, to produce relationship effects for use in dyadic ARRMA partner effects are computed and necessary. We will return to this later.

To reiterate, for each individual the social relations analysis will produce actor and partner components (equations 1 and 2) of interpersonal responses and metaperceptions. Only the actor and partner components in reciprocal responses, and the actor components of metaperceptions are relevant for individual level ARRMA. Once the relevant actor and partner effects are organized in a new data set, individual level ARRMA parameter estimation commences.

With social relations modeling it is very important to have multiple indicators of a construct so that the relationship component and error of equations 1 and 2 are partitioned. In the absence of multiple indicators, they are confounded. Two details are important. One, multivariate social relations analysis would be used and estimates of latent actor, partner and relationship variance components are produced with two or more indicators. Of the applications available for social relations analysis, only Soremo is capable of accommodating more than two indicators of a construct. Two indicators is the limit in TripleR. Two, the relevant components of the SRM are produced for each indicator of a latent construct. That is, actor and partner components of interpersonal responses, and actor components of metaperceptions are computed for each of the indicators. These indicators as organized in Supplementary Table 2 are the data for minimal ARRMA parameter estimation at the individual level of analysis.

Path and structural equation modeling are appropriate for parameter estimation. When using path modeling, the actor and partner components of the indicators, and the actor components of metaperceptions for the indicators of a latent construct are averaged to estimate the generalized (i.e., individual level) ARRMA parameters displayed in Figure 2 (see Supplementary Material). This path model is just-identified, meaning that there is just enough measured data to estimate assumed reciprocity (path B), reciprocity (path A), and metaperception accuracy (path C). Because the model is just-identified, the chi-square test of ARRMA model fit is precluded. However, as will be discussed later, assessment of model fit is less important than assessment of how the parameters of the model vary in different social psychological contexts.

The path modeling strategy is illustrated using published data from a study of self-referenced interpersonal similarity (Malloy, 2019). Note that all data files and output files for the social relations analyses, path and SEM models in amw format, and all output from analyses are posted in the data archive section of the following website (If requested the address of this website will be provided). Data from 150 dyads in families are used. In 25 four-person round robins, family members rated the extent to which: this person and I are from a similar social class; this person thinks like me; this person treats people like I do; this person is similar to me; and this person behaves like me. Responses were on a 7 point scale (1 completely disagree-7 completely agree). Factor analysis demonstrated that these indicators load on a single latent similarity construct (see McCroskey et al., 2006). Multivariate social relations analysis conducted with Soremo produced latent actor, partner, and relationship variance components based on five indicators. In addition, actor and partner components for each indicator of latent similarity were output by Soremo, along with the actor components of metaperceptions for each of the five indicators. These components were averaged. Then the ARRMA parameters in Figure 2 were estimated. Results of this analysis are presented in Table 2 and show that family members assumed reciprocity (parameter B) of interpersonal similarity quite strongly with a statistically reliable structural coefficient (*b* = 0.924 and β = 0.912, *p* < 0.001). If family members judged other family members as similar or different from themselves, they assumed that those family members reciprocated their similarly judgments. Reciprocity was also substantial; the covariance of family members’ perceiver and target components (parameter A) was 0.721, *p* < 0.001 and *r* = 0.713 when standardized. If one member judged family members as generally similar or different from herself, they judged her similarly. There was no evidence of metaperception accuracy (parameter C) at the individual level; that is, family members were not aware of how similar to themselves others judged them to be, as indicated by a structural coefficient (*b* = 0.066, *p* = 0.203 and β = 0.056) that did not differ reliably from zero.

**TABLE 2:** Individual level ARRMA model: perceived interpersonal similarity in families.

ARRMA parameter	Unstandardized	SE	Standardized	Probability
**Full ARRMA model**				
Assumed reciprocity (B)	0.924	0.044	0.912	<0.001
Reciprocity (A)	0.721	0.125	0.713	<0.001
Metaperception accuracy (C)	0.066	0.052	0.056	0.203
**Restricted individual level ARRMA model**				
Assumed reciprocity (B)	0.964	0.031	0.951	<0.001
Reciprocity (A)	0.721	0.125	0.713	<0.001
Metaperception accuracy (C)	–	–	–	

*χ(1df) = 1.606, p = 0.205.*

*– parameter fixed to zero.*

Because metaperception accuracy was absent, parameter C was constrained to zero and the parameters of the restricted ARRMA model were estimated. Somewhat stronger assumed reciprocity (parameter B) was observed (*b* = 0.964, *p* < 0.001 and β = 0.951) and the estimate of reciprocity (parameter A) did not change, as should be (*r* = 0.721, *p* < 0.001 and β = 0.713). This constraint permitted the computation of a chi-square statistic with one degree of freedom that was χ (1) = 1.606, *p* = 0.205, and showed that constraining metaperception accuracy to zero did not impair model fit. This showed that the joint specification of assumed reciprocity and reciprocity of ARRMA adequately fit the interpersonal similarity judgments in families at the individual level. Both the full and restricted path models explained approximately 91% of the variance in the average actor components of metaperceptions of interpersonal similarity.

Alternatively, structural equation modeling can estimate ARRMA parameters at the individual level as seen in Supplementary Figure 1. The model in Supplementary Figure 1 contains three latent constructs: actor components in similarity judgments (ACTOR), partner components in similarity judgments (PARTNER), and actor components of metaperceptions of similarity judgments (ACTOR_MP). There are five indicators of each construct. Each indicator has an error component; E1 through E5 are error components of indicators of actor components, E6 through E10 are error components of indicators of actor components of metaperceptions, and E11 through E15 are error components of partner effects in similarity judgments. Because a single individual produces similarity judgments and metaperceptions, error components are correlated. Structural coefficients B, A, and C in Supplementary Figure 1 are estimates of assumed reciprocity, reciprocity, and metaperception accuracy, respectively.

Table 3 shows the unstandardized (U) factor loadings of the appropriate SRM components indicating each of the latent constructs, the associated standard errors (SE), and the standardized estimates (S). The factor loading for a marker variable fixed at 1.00 sets the metric of each latent construct; all indicators were in a 7 point metric. All free loadings were reliably different from zero. Table 4 shows the covariances, standard errors, and correlations of the error components of indicators of the latent actor response construct, and the latent actor metaperception construct. Error components of the indicators of these two latent constructs, that statistical theory stipulates should correlate at *r* = 0.00, were positively correlated, and four of five were reliably different from zero with standardized estimates ranging from *r* = 0.374 to *r* = 0.872. Because the same individual made perceptions and metaperceptions, error components of indicators correlated systematically. This dependence of error components is likely to occur for most structural equation models of ARRMA because they are mono-methods of measurement (i.e., produced by one individual). Consequently, error components of indicators of actor response and actor metaperception constructs should be correlated.

**TABLE 3:** Measurement models individual level ARRMA interpersonal similarity construct in families.

	Actor effects			Partner effects			Actor effects_MP		
Indicator	U	SE	S	U	SE	S	U	SE	S
Similar social class	1.00	–	0.581	1.00	–	0.553	1.00	–	0.625
Thinks like me	1.507	0.225	0.889	1.634	0.271	0.881	1.470	0.194	0.924
Treats others as I do	1.404	0.213	0.866	1.598	0.273	0.834	1.213	0.169	0.857
Similar to me	1.562	0.230	0.913	1.757	0.289	0.903	1.366	0.182	0.912
Behaves like me	1.800	0.261	0.934	1.889	0.310	0.906	1.475	0.197	0.910

*U are unstandardized loadings, SE are standard errors, and S are standardized loadings.*

*textit– maker variable that set the metric of the construct with unstandardized loading fixed at 1.00.*

*All free construct loadings p < 0.001.*

**TABLE 4:** Covariances of error components of indicators of latent perceiver effects in similarity judgments and metaperceptions.

Component pairs	Unstandardized	SE	Standardized	Probability
E1-E6	0.892	0.139	0.872	<0.001
E2-E7	0.069	0.038	0.250	0.067
E3-E8	0.223	0.047	0.646	<0.001
E4-E9	0.094	0.036	0.374	0.008
E5-E10	0.115	0.041	0.425	0.005

The results of the structural equation model of ARRMA lead to the same conclusions as the path model, as should be the case. Assumed reciprocity (parameter B) was substantial with *b* = 1.074, *p* < 0.001 and β = 0.961, and the reciprocity covariance (parameter A) was 0.305, *p* < 0.001, and *r* = 0.745 when standardized. As seen previously, metaperception accuracy was weak with *b* = 0.027, *p* = 0.723, and *r* = 0.019. These results are in Table 5. The ARRMA SEM at the individual level explained approximately 95% of the variance in the latent metaperception of similarity construct. When the metaperception accuracy parameter (C) was fixed to 0, the chi-square was χ (83) = 280.495, *p* < 0.001, whereas the chi-square for the full model was χ (82) = 280.375. The chi-square difference was Δχ (1) = 0.12, *p* > 0.05, and as seen in the path modeling, fixing the metaperception accuracy parameter to 0 did not adversely impact model fit. The measurement model is the prime source of the lack of fit of this model. Considering the Bentler-Bonnet normed fit index (NFI), the value for the SEM ARRMA model is 0.86 and is much closer to the fully saturated model (NFI = 1.00) than it is to the fully independent model (NFI = 0). Both path modeling and SEM produced an identical pattern of results for the ARRMA phenomena, and explained a very substantial portion of the variance in the latent endogenous metaperception of similarity construct. The models developed are for indistinguishable dyads. With distinguishable dyads (e.g., female and male), the path or structural equation models can be run using a multiple groups analysis to assess if the individual level ARRMA model fits the data equally well for members of each category.

**TABLE 5:** SEM structural coefficients: individual level ARRMA model.

Parameter	Unstandardized	SE	Standardized	Probability
B	1.074	0.092	0.961	<0.001
A	0.305	0.083	0.745	<0.001
C	0.027	0.076	0.019	0.723

*Parameter B is assumed reciprocity, A is reciprocity, and C is metaperception accuracy.*

Individual level ARRMA focuses on individuals’ assumptions about others’ responses to them, the actual reciprocity in dyadic interactions, and their ability to know accurately others’ responses to them. The focus is on the assumptive and predictive responses of one to many, and the actual responses of many to the one. Dyadic ARRMA is concerned with these processes in specific dyadic arrangements, with a focus on the assumptive and predictive responses of one to a specific other, and the actual responses of the specific other to the one.

### ARRMA Parameter Estimation at the Dyadic Level

Dyadic ARRMA requires attention to details that are not a concern at the individual level. In some cases, data may be incomplete with only one member of the dyad providing a response, whereas the other does not. Fortunately, even in the case of incomplete data there can be sufficient information to estimate the parameters of the minimal dyadic ARRMA. Another concern is with the distinguishability of dyad members. That is, can the dyad members be distinguished on a variable that is relevant to an outcome of interest; in ARRMA that outcome is a metaperception.

#### Minimal Dyadic ARRMA With Incomplete Data

Imagine a study where 4 members of an in-group (A through D) interact with 4 members of an out-group (W through Z), and only intergroup responses are made; in-group responses are not made by those in either group. Because group membership differentiates the dyad members, analytic methods for distinguishable dyads can be used (Griffin and Gonzalez, 1995). These data conform to an asymmetric block structure with reciprocal measurements. However, because only members A through D of the in-group predict out-group members’ responses to them; these metaperceptions conform to a half-block structure with unidirectional measurements (Malloy, 2018b). To elaborate the approach to dyadic ARRMA with reciprocal interpersonal responses and unidirectional metaperceptions, again consider the hypothetical data in Table 2. The interpersonal responses conform to an asymmetric block structure, and the metaperceptions conform to a half-block structure. Social relations analysis of each structure can be accomplished using an application called Blocko (Kenny and Xuan, 2006), and separate analyses would be conducted for interpersonal responses and metaperceptions. Then the appropriate SRM components from these analyses would be output, and then a new data set formed for estimation of the minimal dyadic ARRMA model.

To begin, Blocko is used to conduct a social relations analysis of the data from the asymmetric block design. That analysis would produce the SRM components of equations 1 and 2 for in-group members’ responses to the out-group, and for the out-group members’ responses to the in-group. Blocko would also be used to analyze the data from the half-block structure of in-group metaperceptions of out-group members’ judgments of them. Using output from these analyses, the SRM relationship effect estimates for reciprocal intergroup responses (i.e., in-group to out-group and vice versa) must be computed. One would instruct Blocko to output the raw data, as well as actor and partner effect estimates with the grand mean removed. The grand mean for each of *k* groups is included with standard output from Blocko. Each individual’s raw data (*X*_*ijk*_), individual level components (actor α*_*ik*_* and partner β*_*jk*_*), and the grand mean for each group (μ*_*k*_*) would be merged into a new data set. Then the following calculations would be completed for the 16 responses of the in-group to the out-group, and for the 16 responses of the out-group to the in-group in each of the asymmetric blocks.


(24) $$X_{ijk}-\mu_{k}-\alpha_{ik}-\beta_{jk}=\gamma_{ijk}$$

Equation 24 yields the relationship effect estimates for *i*’s dyadic response to *j* on variable *X* in group *k*. Because only in-group members predict out-group members’ responses to them, a similar calculation is performed for in-group members’ unidirectional metaperceptions (*MP*). This yields the relationship effect estimates (γ_*mp,ijk*_) quantifying in-group member *i*’s prediction of out-group member *j*’s response to *i*. That is:


(25) $$MP_{ijk}-\mu_{k}-\alpha_{ik}-\beta_{jk}=\gamma_{mp,ijk}$$

Following this, the reciprocal relationship effect estimates (i.e., gammas) of intergroup responses (i.e., γ*_*ijk*_* and γ*_*jjk*_*) would be merged with unidirectional (i.e., in-group to out-group only) relationship effect estimates in metaperceptions (γ*_*mp*,ijk_*). These three measurements would each appear on a single row of a new data set called a *dyad structure*. The minimal dyadic ARRMA is displayed as a path model in Figure 3, and estimation of the model’s parameters can be produced using path analysis or structural equation modeling. The path model is just-identified with sufficient information to estimate the ARRMA parameters, but insufficient information to assess model fit.

#### Minimal Dyadic ARRMA With Distinguishable Members

**FIGURE 4:**
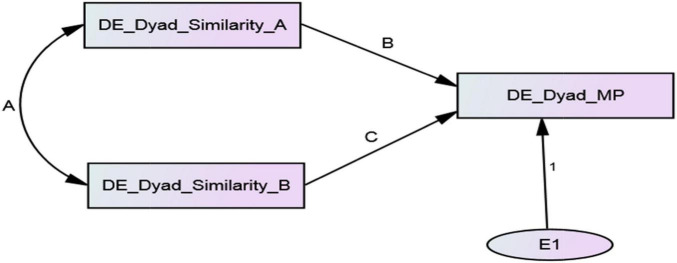
Minimal dyadic ARRMA with double data entry with distinguishable dyads.

The ARRMA parameters (B: assumed reciprocity, A: reciprocity, and C: metaperception accuracy) with distinguishable dyad members can also be estimated with the minimal dyadic ARRMA model when double data entry (i.e., pairwise data entry) is used (see Figure 4). Presumed for this example is that dyad members in families are distinguishable, although their specific role or position in their families was unknown. Displayed in Supplementary Table 3 is the double entry, pairwise data set. Double entry, pairwise entry means that the data for each dyad member, on each variable, are entered twice in the pattern displayed in Supplementary Table 3. All unstandardized estimates for the minimal dyadic ARRMA with distinguishable members are reliably different from zero and document assumed reciprocity (*b* = 0.705, *p* < 0.001 and β = 0.697), reciprocity (*covariance* = 0.102, *p* < 0.001 and *r* = 0.392), and metaperception accuracy (*b* = 0.162, *p* < 0.001 and β = 0.60) for interpersonal similarity judgments in families. The minimal dyadic ARRMA explained about 60% of the variance in dyadic metaperceptions, and these estimates are summarized in Table 6.

**TABLE 6:** Minimal dyadic ARRMA for interpersonal similarity in families: double entry data input with assumed dyad distinguishability.

Parameter	Unstandardized	SE	Standardized	Probability
B	0.705	0.042	0.697^+^	<0.001
A	0.102	0.023	0.392	<0.001
C	0.162	0.042	0.160	<0.001

*These estimates are for self-referenced judgments of interpersonal similarity in dyads within families. B is assumed reciprocity, A is reciprocity, and C is metaperception accuracy.*

*^+^This estimate was reported inaccurately (0.353) in Malloy (2019, Table 6), however the unstandardized estimate, standard error, and probability were reported correctly.*

#### Full Dyadic ARRMA With Distinguishable Members

**FIGURE 5:**
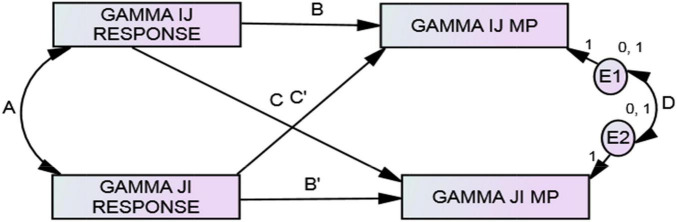
Full dyadic ARRMA model with distinguishable dyad members.

The full dyadic ARRMA model is estimated when there are reciprocal interpersonal responses and metaperceptions collected from each member of the dyad, and dyad members are distinguishable. An example of distinguishable dyads is a study of the verbal interaction of mothers and 28-month-old toddlers (Malloy et al., 2022); dyad members were distinguishable developmentally, and this had an effect on the complexity of their verbal behavior. The full ARRMA model with distinguishable dyads is displayed in Figure 5. To estimate the parameters of the full dyadic ARRMA with distinguishable members, dyad input of SRM relationship effects described previously would be used, and the model is just-identified. There are two estimates of assumed reciprocity (parameters B and B’), one estimate of reciprocity (parameter A), two estimates of metaperception accuracy (C and C’), and one estimate of the reciprocity of metaperceptions (parameter D). Note that parameter D is not estimated when the minimal dyadic ARRMA with distinguishable members is modeled.

For this analysis, family members were assumed to be distinguishable because they always occupy different roles, unless they are monozygotic twins. That is, their family roles are not interchangeable. Table 7 shows the parameter estimates for the full dyadic ARRMA model of perceived interpersonal similarity with distinguishable family members displayed as a path model in Figure 4. All parameter estimates in the full dyadic ARRMA model are reliably different from 0. The two estimates of assumed reciprocity (B and B’) were *b* = 0.625, *p* < 0.001 (β = 0.668) and *b* = 0.812, *p* < 0.001 (β = 0.738), respectively were statistically reliable, and showed that dyad members assumed that their judgments of family members unique similarity to them were reciprocated. The single covariance estimating reciprocity (A) was 0.102, *p* < 0.001 (*r* = 0.395) and was reliable statistically; this showed that unique interpersonal similarity judgments were, in fact, reciprocated. If one member of the dyad judged the other member as uniquely similar or dissimilar, the other member reciprocated this judgment. Reciprocity was weaker than assumed reciprocity. The two estimates of metaperception accuracy (C and C’) were *b* = 0.149, *p* = 0.003 (β = 153) and *b* = 0.166, *p* = 0.009 (β = 0.156), respectively; both were statistically reliable and showed that family members were accurately aware of others’ unique judgments of their interpersonal similarity. The reciprocity of metaperceptions was estimated by the covariance of the disturbances in the two metaperception constructs. This covariance estimating parameter D was statistically reliable (−0.029, *p* = 0.001, and *r* = −0.277), and showed that family members’ predictions of specific others unique similarity/dissimilarity to themselves (plus error) were related inversely. The full ARRMA model explained approximately 55 and 66% of the variance in dyadic metaperceptions.

**TABLE 7:** Full dyadic ARRMA for interpersonal similarity in families: dyad input with assumed dyad distinguishability.

Parameter	Unstandardized	SE	Standardized	Probability
B	0.625	0.056	0.668	<0.001
B’	0.812	0.058	0.738	<0.001
A	0.102	0.223	0.395	<0.001
C	0.149	0.051	0.153	0.003
C’	0.166	0.063	0.156	0.009
D	−0.029	0.009	−0.277	0.001

*B and B’ are assumed reciprocity, A is reciprocity, and C and C’ are metaperception accuracy, and D is the reciprocity of metaperception. R^2^ MP = 0.55 and R^2^ MP’ = 0.66. χ(0) = 0.*

#### Full Dyadic ARRMA, Indistinguishable Dyads

Estimation of the dyadic ARRMA parameters is much more complicated when the dyad members are indistinguishable. In a previous example of in-group and out-group responses, dyad members were distinguishable based on group membership. In another, adult women and children were distinguishable developmentally. In contrast, estimation of dyadic ARRMA parameters when dyad members are indistinguishable requires specific constraints on parameters, and adjustments of estimates of model fit. The reason for these constraints and adjustments is that designation of dyad members as *i* or *j* within a dyad is arbitrary (Olsen and Kenny, 2006). This means that dyad members cannot be differentiated on a variable (e.g., sex, group membership, status) that impacts a dyadic metaperception. Because the dyad members are indistinguishable (or interchangeable), designation as *i* or *j* is arbitrary, and this fact imposes implicit statistical constraints on the model. In effect, these constraints force the analysis to the dyadic level, although *i*’s and *j*’s unique interpersonal responses to one another are variables in the model. Different data arrangements can be used to estimate the parameters of dyadic ARRMA with indistinguishable members; this is, dyad input or the double entry, pairwise methods may be used. Each data structure has implications for ARRMA parameter estimation. For reasons elaborated later, there is an advantage to the double entry method of data organization. For this example, double (i.e., pairwise) data entry (see Supplementary Table 3) was used, and SRM relationship effect estimates for five indicators of an interpersonal similarity construct, and the five indicators of a metaperceived similarity construct from an initial social relations analysis were averaged.

**FIGURE 6:**
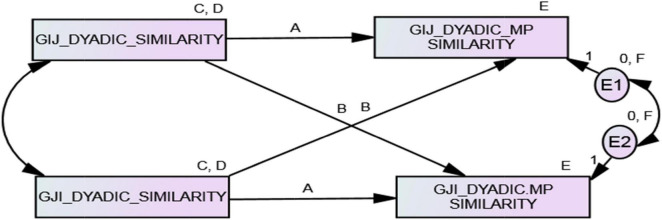
Full dyadic ARRMA for indistinguishable dyads with Olsen and Kenny (2006) constraints. G indicates an SRM relationship effect (gamma).

Olsen and Kenny (2006) specify implicit constraints on model parameters when dyad members are indistinguishable. The full dyadic ARRMA model is appropriate in this case and is presented in Figure 6, with the Olsen-Kenny constraints represented by letters and numbers. Those that are the same indicate an equality constraint imposed on the model. They are:

$\bar{X}$_γ*ij*_ = $\bar{X}$_γ*ji*_ (equal predictor means: C)

*S^2^_γ*ij*_* = *S^2^_γ*ji*_* (equal predictor variances: D)

*M_γ *i,mp,j*_* = *M_γ*j,mp,i*_* (equal outcome intercepts: E)

*S*^2^_*d*1_ = *S*^2^_*d*2_ (equal disturbance variances: F)

*d* = *d’* (equal disturbance effects on the outcome: 1.00)

#### Implications for Parameter Estimation

The implicit constraints specified by Olsen and Kenny (2006) must be instituted when estimating dyadic ARRMA parameters with indistinguishable members. Olsen and Kenny discuss three classes of models when illustrating the implications of these constraints; most relevant to ARRMA is the APIM (Kenny, 1996; i.e., actor-partner interdependence model). ARRMA and APIM are structurally equivalent although the psychological meanings of their parameters are not. Typically, the APIM is deployed with a single interaction dyadic design when individuals are only in one dyad (Malloy and Albright, 2001). Dyad members are typically distinguishable. For each dyad member, two variables are measured; for example, *X* and *Y*. Dyad member 1 has scores *X*_1_ and *Y*_1_, and dyad member 2 has scores *X*_2_ and *Y*_2_. The direct effect of *X*_1_ on *Y*_1_, and the direct effect of *Y*_1_ on *Y*_2_ are termed actor effects in APIM, and the effects of *X*_1_ on *Y*_2_ and *X*_2_ on *Y*_1_ are termed partner effects. An estimate of the covariance of *X*_1_ and *X*_2_ is produced by the covariance of the disturbance effects on *Y*_1_ and *Y*_2_. In contrast, estimation of ARRMA presumes a multiple interaction design in which multiple actors interact with multiple partners. Additionally, estimates of dyadic ARRMA parameters with indistinguishable members is at the level of the dyad. Typically, APIM parameter estimates are at the individual level of analysis, although Kenny (2017) has specified an APIM using relationship effect estimates from a social relations analysis of data from a round robin design. The actor effects in APIM are individual level effects of each members’ scores on *X*_1_ and *X*_2_ (e.g., marital satisfaction) on their scores on *Y*_1_ and *Y*_2_ (e.g., marital commitment). In contrast, these same parameters in the full dyadic ARRMA quantify dyadic assumed reciprocity. The partner effects in APIM are interpersonal; they are the effects of *X*_1_ on *Y*_2_ and *X*_2_ on *Y*_1_. For example, does one member’s marital satisfaction (e.g., *X*_1_) affect the partner’s marital commitment (e.g., *Y*_2_) and vice versa. In ARRMA, these interpersonal parameters quantify dyadic metaperception accuracy; that is, do dyad members’ know accurately the unique responses that specific partners in different dyads make to them? In APIM the covariance of the causal or temporally precedent variables (i.e., *X*_1_ and *X*_2_) quantify their association, whereas in ARRMA this covariance quantifies dyadic reciprocity. In APIM disturbances in endogenous variables are correlated and quantify an intraclass (i.e., within-dyad) covariance of an outcome (Olsen and Kenny, 2006), whereas in ARRMA this covariance quantifies reciprocity of dyadic metaperceptions. This shows that despite their structural similarity, ARRMA and APIM parameters quantify different phenomena in different dyadic interaction contexts (multiple vs. single interaction designs, respectively). Despite these differences, analytic methods for APIM and ARRMA parameter estimation with indistinguishable dyads require the same constraints specified by Olsen and Kenny (2006).

#### Estimation of Full Dyadic ARRMA Parameters

Presented in Figure 6 is the full dyadic ARRMA model for indistinguishable members with the Olsen-Kenny constraints. The analysis begins with the family members’ SRM relationship effect estimates when judging the similarity of specific family members to themselves, and these data were entered using the double entry, pairwise method. The Olsen-Kenny constraints yield what is referred to as the I-SAT (indistinguishable, saturated) model. The chi-square for the I-SAT is a function of which dyad member is designated as 1 or 2, and is used to adjust the degrees of freedom of the chi-square testing the fit of the ARRMA model. With the double entry, pairwise data structure used here, the χ^2^ produced for the dyadic ARRMA while ignoring the implicit constraints on the model, is equal to the I-SAT χ^2′^ (see Olsen and Kenny, 2006, p. 134). In the present case, χ(6) = 0. If the data were in a dyad format, the chi-square and degrees of freedom for the I-SAT would be subtracted from the chi-square and degrees of freedom for the unconstrained ARRMA model. Olsen and Kenny (2006) provide detailed guidance on how to do this.

The estimates of dyadic ARRMA parameters from the unconstrained ARRMA model and the I-SAT ARRMA model with Olsen-Kenny constraints (see Figure 6) are presented in Table 7. All estimates of ARRMA parameters in the I-SAT, and in the unconstrained ARRMA model, are reliably different from zero, and documents that assumed reciprocity (B) in the I-SAT (*b* = 0.705, *p* < 0.001), reciprocity (A) in the I-SAT (covariance = 0.102, *p* < 0.001), and meta-accuracy (C) in the I-SAT (*b* = 0.162, *p* < 0.001) all occur reliably when family members make dyadic similarity judgments. That is, family members assume that if they judge other family members as uniquely similar or dissimilar to themselves, those family members reciprocate these unique judgments. In fact, unique similarity judgments were reciprocated. Family members also knew accurately if specific family members judge them as uniquely similar or dissimilar to themselves. And finally, there was evidence for reliable reciprocity of similarity metaperceptions (I-SAT covariance = −0.0295, *p* < 0.001 with *r* = −0.277); if one member predicts that another judges him or her as uniquely similar or dissimilar, the other tends to predict inversely.

In the unconstrained model, there are two estimates of assumed reciprocity and metaperception accuracy, whereas with Olsen-Kenny constraints these parameters are constrained to equality (see Table 8). Notice that the values of the parameters constrained to equality using the Olsen-Kenny method are equal to the average of the unstandardized parameter estimates in the unconstrained model within rounding error (see Table 7).

**TABLE 8:** Full dyadic ARRMA with indistinguishable dyads: unconstrained and Olsen-Kenny constraints.

	Unconstrained model			Olsen-Kenny constraints			
Parameter	Unstandardized	SE	Standardized	Unstandardized	SE	Standardized	*p*
B	0.625	0.056	0.668	0.705	0.042	0.697	<0.001
B’	0.812	0.057	0.738	0.705	0.042	0.697	<0.001
R	0.102	0.023	0.395	0.102	0.023	0.392	<0.001
C	0.149	0.051	0.153	0.162	0.042	0.160	<0.001
C’	0.166	0.063	0.156	0.162	0.042	0.160	<0.001
R_MP	−0.0295	0.009	−0.277	−0.0295	0.009	−0.275	<0.001

*B and B’ are assumed reciprocity, R is reciprocity, C and C’ are metaperception accuracy, and R_MP is reciprocity of disturbances (E1 and E2) in metaperceptions.*

### ARRMA and Model Fit

Model fit is concerned with the isomorphism of the variance/covariance matrix implied by a model, and the observed variance/covariance matrix for the variables in the model. A theoretical model that perfectly fits the data reproduces exactly the observed variance/covariance matrix. With ARRMA, the issue of model fit is less important than understanding how the three phenomena vary in different social contexts (e.g., strangers vs. highly acquainted) and for different classes of interpersonal behavior (e.g., trait judgments, affect, behavior). One should not expect ARRMA to adequately fit the data in all cases; rather, there are social psychological contexts when ARRMA should fit the data, and contexts when it should not. Consider assumed reciprocity; this is the belief that others generally or specific others think, feel, and intend to behave toward us as we think, feel, and intend to behavior toward them. Kenny (1994) showed that assumed reciprocity is substantial for interpersonal affect, but non-existent for trait judgments at the individual and dyadic levels. Consequently, this parameter of ARRMA should vary for different types of dyadic responses. Also consider the reciprocity of trait perceptions at the individual and dyadic levels; meta-analysis indicates this reciprocity is essentially zero expressed as a correlation (Kenny, 1994). For interpersonal affect (e.g., attraction), Kenny (1994) reported that estimates of reciprocity at the individual level are often near zero; among those who are very highly acquainted, generalized reciprocity is moderate (see also Wright et al., 1984; Malloy, 2018a). Meta-analytic estimates show that dyadic reciprocity is weak to moderate among people who meet for the first time, but this rises to about *r* = 0.61, on average, among those who are well acquainted (Kenny, 1994). Meta-accuracy is awareness of others’ or specific other’s responses to oneself, and again, there is moderation by the nature of the judgment task. When the focus is on traits (Kenny, 1994) or academic ability (Malloy et al., 2007), generalized meta-accuracy is substantial; whereas dyadic meta-accuracy is near zero. This means that people know accurately how others generally judge their traits and ability, but are less veridical regarding specific others’ judgments. People are also accurately aware of how attracted to them others are, but are less accurate when predicting a specific others’ unique attraction to them (Kenny, 1994; Malloy, 2018a).

As these patterns show, the ARRMA phenomena are likely to vary in different contexts and for different classes of interpersonal responses; therefore, the model should not be expected to adequately fit the data in all of them. Rather, the focus should be on when ARRMA does and does not fit the data; that is, how does variation of the social context and the nature of interpersonal responses moderate ARRMA phenomena? Because there should be moderation of ARRMA parameters in different social contexts and for different interpersonal responses, multiple groups analysis can be used to assess if ARRMA fits the data equally well or less well in them.

## Summary and Conclusion

Theories of interpersonal behavior (Sullivan, 1939, 1949; Leary, 1957; Schutz, 1966; Carson, 1969; Kiesler, 1983) propose that the behavior of one member of a dyad signals what reciprocal response by the other member is appropriate and breeds reciprocity or complementarity (Sadler and Woody, 2003). In dyads, people are aware of their own cognitions, affect, and behavior (Tversky and Kahneman, 1973), and are simultaneously motivated to know how the other will respond to them (Laing et al., 1966). Knowing the other’s thoughts, feelings and behavioral intentions is a core social motive (Fiske, 2014), and people are inclined to assume that others’ responses to them will match their responses to those others (Heider, 1958; Tagiuri, 1958). ARRMA is an explicit recognition of the simultaneity of assumed and actual interpersonal behavior built upon the componential structure of the social relations model. Consistent with the logic of social relations modeling, ARRMA phenomena operate in general at the individual level, and in specific dyadic arrangements. ARRMA integrates phenomena studied independently of one other in the past. Most generally, ARRMA is an integrative theoretical model of assumed and actual interpersonal responses in dyads, and can answer the question: In dyadic interaction, do people share a common interpersonal reality?

## Data Availability Statement

Publicly available datasets were analyzed in this study. This data can be found here: http://thomasemalloy.org/archived-published-data/.

## Ethics Statement

The studies involving human participants were reviewed and approved by the Rhode Island College Institutional Review Board. The patients/participants provided their written informed consent to participate in this study.

## Author Contributions

TM was responsible for all the content of this research.

## Conflict of Interest

The author declares that the research was conducted in the absence of any commercial or financial relationships that could be construed as a potential conflict of interest.

## Publisher’s Note

All claims expressed in this article are solely those of the authors and do not necessarily represent those of their affiliated organizations, or those of the publisher, the editors and the reviewers. Any product that may be evaluated in this article, or claim that may be made by its manufacturer, is not guaranteed or endorsed by the publisher.

## Footnotes

1 The social relations model is a mathematical model of the determinants of dyadic behavior. The determinants are at the individual level of analysis (i.e., actor, partner) and simultaneously at the level of the dyad (i.e., relationship). Relevant components are variables in the ARRMA model. The SRM will be discussed fully later.

## Supplementary Material

Supplementary Figure 1 A latent variable model of ARRMA at the individual level.
