# Supplementary material for: ARRMA: An Integrative Theoretical and Mathematical Model of Assumed and Actual Dyadic Behavior
Source: Front Psychol. 2022 Jun 7;13:834796. doi: 10.3389/fpsyg.2022.834796 (PMC9210992; doi:10.3389/fpsyg.2022.834796)
Supplement: Supplementary file 1 [file Table_1.docx]

Supplemental Material 1

Reorganization of SRM Components for Individual Level ARRMA Parameter Estimation

SRM Components

Round Robin Individual Actor_r Partner_r Actor_mp

1 1 *α_11._ β_.11_ α_mp_11_._*

1 2 *α_12._ β_.12_ α_mp12._*

1 3 *α_13._ β_.13_ α_mp13._*

1 4 α_14._ *β_.14_ α_mp14._*

*n* 1 *α_n1._ β_.n1_ α_mpn1._*

*n* 2 *α_n2._ β_.n2_ α_mpn2._*

*n* 3 *α_n3._ β_.n3_ α_mp3n._*

*n* 4  *α_n4._ β_.n4_ α_mpn4._*

Note. Data for round robin 1 and n. The first subscript is round robin and the second is

individual within round robins. Actor_r and Partner_r are individuals’ actor and partner

components of interpersonal responses. Actor_mp are individuals’ actor components of

metaperceptions (i.e., predictions) of partners’ responses to them.
