# Supplementary material for: ARRMA: An Integrative Theoretical and Mathematical Model of Assumed and Actual Dyadic Behavior
Source: Front Psychol. 2022 Jun 7;13:834796. doi: 10.3389/fpsyg.2022.834796 (PMC9210992; doi:10.3389/fpsyg.2022.834796)
Supplement: Supplementary file 2 [file Table_2.docx]

Supplementary Material 2

Asymmetric Data Structures with Reciprocal, Unidirectional, and Missing Data

Reciprocal Responses Unidirectional Metaperceptions

Out-Group Members Out-Group Members

In-Group Members W X Y Z W X Y Z

A *r_11_ r_12_ r_13_ r_14_ mp_11_ mp_12_ mp_13_ mp_14_*

B *r_21_ r_22_ r_23_ r_24_ mp_21_ mp_22_ mp_23_ mp_24_*

C *r_31_ r_32_ r_33_ r_34_ mp_31_ mp_32_ mp_33_ mp_34_*

D *r_41_ r_42_ r_43_ r_44_ mp_41_ mp_42_ mp_43_ mp_44_*

Reciprocal Responses Missing Metaperceptions

In-Group Members In-Group Members

Out-Group Members A B C D A B C D

W *r_11_ r_12_ r_13_ r_14_* -- -- -- --

X *r_21_ r_22_ r_23_ r_24_* -- -- -- --

Y *r_31_ r_32_ r_33_ r_34_* -- -- -- --

Z *r_41_ r_42_ r_43_ r_44_* -- -- -- --

Note. Responses by A through D to W through Z labeled *r,* are in-group responses to the out-group; those labeled *mp* are metaperceptions. Responses by W through Z to A through D labeled *r,* are out-group responses to the in-group.

-- data not collected
