# Supplementary material for: ARRMA: An Integrative Theoretical and Mathematical Model of Assumed and Actual Dyadic Behavior
Source: Front Psychol. 2022 Jun 7;13:834796. doi: 10.3389/fpsyg.2022.834796 (PMC9210992; doi:10.3389/fpsyg.2022.834796)
Supplement: Supplementary file 3 [file Table_3.DOCX]

Supplementary Material 3

Double or Pairwise Data Entry for Three Dyads

Dyad *X X’ Y Y’*

1 *X_11_ X’_12_ Y_11_ Y’_12_*

1 *X’_12_ X_11_ Y’_12_ Y_11_*

2 *X_21_ X’_22_ Y_21_ Y’_22_*

2 *X’_22_ X_21_ Y’_22_ Y_21_*

3 *X_31_ X’_32_ Y_31_ Y’_32_*

3 *X’_32_ X_31_ Y’_32_ Y_31_*

Note. X and Y are scores for one member of the dyad, while X’ and Y’ are scores for the other member of the dyad. The first subscript is dyad and the second is person within dyad.
