# Supplementary figures and images for: ARRMA: An Integrative Theoretical and Mathematical Model of Assumed and Actual Dyadic Behavior
Source: Front Psychol. 2022 Jun 7;13:834796. doi: 10.3389/fpsyg.2022.834796 (PMC9210992; doi:10.3389/fpsyg.2022.834796)

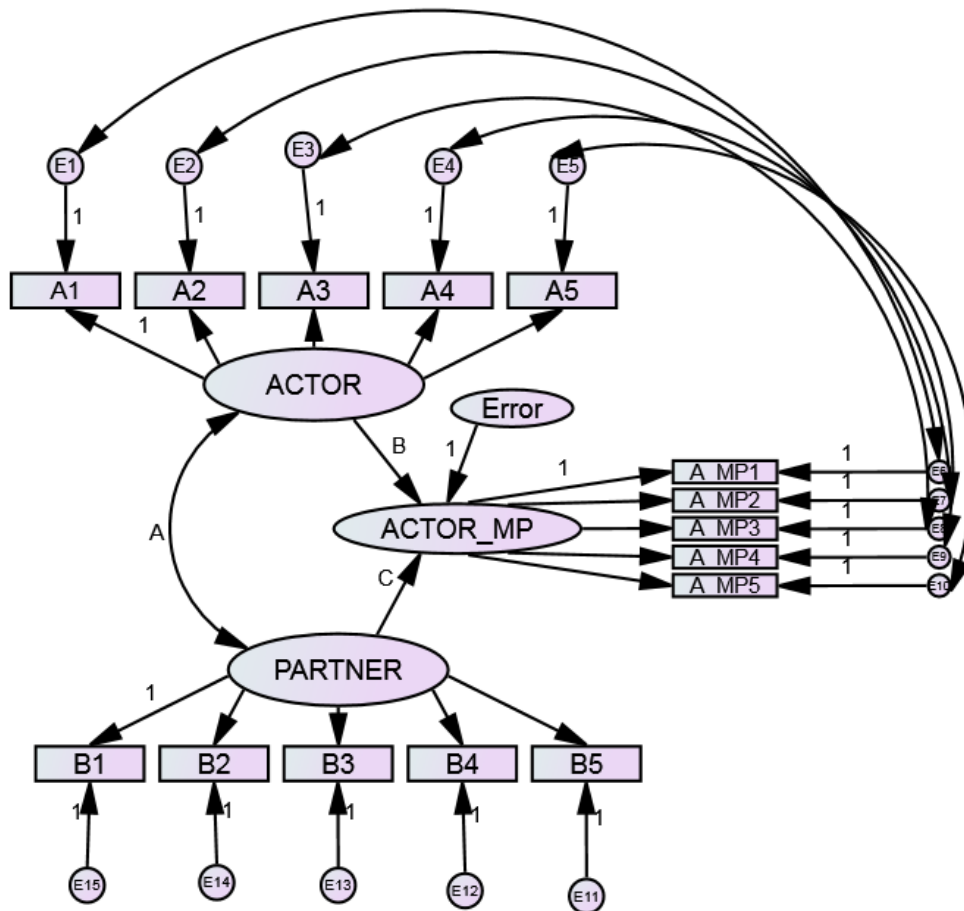

Supplement: Supplementary Figure 1 — A latent variable model of ARRMA at the individual level. [file Image_1.pdf]
